# A comprehensive analysis focusing on cuproptosis to investigate its clinical and biological relevance in uterine corpus endometrial carcinoma and its potential in indicating prognosis

**DOI:** 10.3389/fmolb.2022.1048356

**Published:** 2022-12-07

**Authors:** Qihui Wu, Ruotong Tian, Hong Tan, Jiaxin Liu, Chunlin Ou, Yimin Li, Xiaodan Fu

**Affiliations:** ^1^ Department of Obstetrics and Gynecology, Xiangya Hospital, Central South University, Changsha, China; ^2^ National Clinical Research Center for Geriatric Disorders, Xiangya Hospital, Changsha, China; ^3^ Department of Pharmacology, School of Basic Medical Sciences, Shanghai Medical College, Fudan University, Shanghai, China; ^4^ Department of Pathology, Second Xiangya Hospital, Central South University, Changsha, China; ^5^ Department of Pathology, School of Basic Medical Sciences, Central South University, Changsha, China; ^6^ Department of Pathology, Xiangya Hospital, Central South University, Changsha, China; ^7^ Department of Pathology, Fudan University Shanghai Cancer Center, Shanghai, China; ^8^ Department of Oncology, Shanghai Medical College, Fudan University, Shanghai, China

**Keywords:** endometrial carcinoma, cell death, cuproptosis, immune microenvironment, prognosis signature

## Abstract

Cuproptosis, a novel copper-dependent cell death involving mitochondrial respiration, is distinct from other known death mechanisms, which inspires us to study further in uterine corpus endometrial carcinoma (UCEC). Herein, leveraging comprehensive data from TCGA-UCEC, we conducted transcriptional and genetic analyses of 13 recently identified cuproptosis genes. We discovered severe genetic instability of cuproptosis genes, extensive positive correlations among those genes with each other at the mRNA level, and their involvement in oncogenic pathways in UCEC samples. Next, WGCNA was performed to identify a potential module regulating cuproptosis, in which the hub genes, in addition to 13 cuproptosis genes, were drawn to construct a scoring system termed Cu. Score. Furthermore, its clinical and biological relevance and tumor immune landscape, genetic alterations, as well as predicted sensitivity of chemotherapy drugs in different Cu. Score subgroups had been discussed extensively and in detail. Additionally, univariate Cox and LASSO regression were performed to identify 13 cuproptosis-related prognostic genes to establish a prognostic signature, the Risk. Score. Integrating the Risk. Score and clinical parameters, we established a nomogram with excellent performance to predict the 1-/3-/5-year survival probabilities of UCEC patients. To conclude, we conducted a comprehensive analysis encompassing cuproptosis and developed a cuproptosis scoring system and a prognostic prediction model for UCEC, which may offer help with individualized assessment and treatment for UCEC patients from the perspective of a novel death mechanism.

## Introduction

Uterine corpus endometrial carcinoma (UCEC) is one of the most prevalent types of gynecological cancers worldwide. According to epidemiological data, the global incidence of endometrial carcinoma has increased steadily over the last decade. There are estimated to be 417,000 new cases and 97,000 deaths worldwide in 2020 (Wang et al., 2020; Sung et al., 2021). Despite advancements in medical devices and treatments, endometrial carcinoma deaths have continued to rise over the past decade. The uncertainty of recurrences and prognoses still confuses clinicians (Lu and Broaddus, 2020; Abu-Zaid et al., 2021). To evaluate the prognosis of endometrial carcinoma patients, traditional risk assessment methods are far from sufficient. Prognostic factors for patients with endometrial carcinoma are currently mainly based on clinical variables such as age, FIGO stage, and pathological subtype. Researchers have shown that certain genetic and molecular factors can also affect the endometrial carcinoma prognosis (Cancer Genome Atlas Research et al., 2013; Bell and Ellenson, 2019). As the pathogenesis and clinical manifestations of endometrial carcinoma are heterogeneous (Gupta, 2017), an effective prognosis prediction method that combines genetic as well as transcriptome alterations with the clinical characteristics of patients with endometrial carcinoma is urgently needed.

As a catalytic cofactor for essential enzymes involved in oxygen transport and energy metabolism, Copper (Cu) exerts paramount effects in providing basic functions for cell survival (Kim et al., 2008). Generally, the concentration of copper in cells is so subtly regulated by metabolic demands and variations of the cellular environment that unusual concentrations bring significant damages to cells (Rae et al., 1999). A previous study suggested that patients with endometrial cancer exhibited lower Cu levels than those of controls. It was found that ever use of intrauterine devices containing Cu was inversely associated with endometrial cancer risk, independent of known risk factors (Felix et al., 2015). However, the mechanism behind it is still unclear. A recent study has shown that copper toxicity-mediated cell death is different from other forms of cell death, and this novel mechanism is termed cuproptosis (Tsvetkov et al., 2022). Cuproptosis occurs when copper binds directly to the aliphatic component of the tricarboxylic acid (TCA) cycle. It leads to the aggregation of lipoacylated proteins and the loss of iron-sulfur cluster proteins, which may ultimately result in cell death due to toxic effects (Tsvetkov et al., 2022). These findings suggest a novel perspective for investigating the application of cuproptosis in cancer treatment (Wang et al., 2022a).

The tumor microenvironment (TME) is a complex system composed of mesenchymal cells, immune cells, extracellular matrix molecules, and inflammatory mediators that determine tumor progression and clinical outcome (Mao et al., 2021). Previous studies have suggested a positive correlation between immune and stromal scores and the clinical characteristics and outcomes of UCEC (Chen et al., 2020; Zhao et al., 2021), and several genes manipulating the immune environment of UCEC can be used to predict prognosis (Ma et al., 2020). Emerging evidence suggests that copper deficiency adversely affects immune function and enables the organism to be susceptible to microbial infection (Munoz et al., 2007). Copper plays an indispensable role in tumor immunity and antitumor therapy (Percival, 1998; Prajapati et al., 2020), and intratumoral copper can regulate PD-L1 expression and affect tumor immune escape (Voli et al., 2020). Recently, some researchers have investigated the relationships between cuproptosis and the immune environment of bladder cancer, glioma, and head and neck squamous carcinomas (Wang et al., 2022b; Song et al., 2022; Tang et al., 2022).

By far, most studies only focus on a few genes involved in cuproptosis. So, there are limited new findings that deserve further exploration. In addition to the 13 cuproptosis genes mentioned in the literature (Tsvetkov et al., 2022), to expand our horizons in cuproptosis, we employ the WGNCA to identify potential cuproptosis-related modules and genes, as well as their associations with the immune microenvironment of endometrial tumors. Also, we developed a cuproptosis scoring system (Cu.Score) and a prognosis prediction gene panel (Risk.Score) for UCEC potential cuproptosis-related modules and genes that were identified by WGCNA. As for the Cu. Score, its relevance to clinical characteristics, immune modulation in the UCEC microenvironment, genetic alterations, and its possibility to guide chemotherapeutic drug selection were comprehensively analyzed. To accurately predict the prognosis of UCEC based on cuproptosis, cuproptosis-related genes with prognostic significance were selected to construct the Risk. Score which exhibited excellent performance in predicting the survival of UCEC patients.

## Materials and methods

### Data source and preprocessing

RNA sequencing data (Fragments per kilobase million, FPKM), somatic mutations, the copy number alteration (CNA), and relevant prognostic and clinicopathological data of UCEC patients were downloaded from the UCSC Xena browser (https://xena.ucsc.edu/public-hubs) and cBioPortal (http://www.cbioportal.org/datasets). The FPKM values were transformed into transcripts per kilobase million (TPM) values and further transformed using a log-2 transformation. Patients with no information on their survival were removed. In this study, 558 samples were included, including 523 tumor samples and 35 normal samples. After that, 523 patients from the TCGA-UCEC cohort were randomly assigned to a training cohort (*n* = 262) and a validation cohort (*n* = 261) in a 1:1 ratio *via* the R package “caret”. Secondly, the expression data of GSE17025 was downloaded from the Gene Expression Omnibus (GEO) and used for subsequent validation. The Human Protein Atlas (HPA) database was used to analyze the protein expression levels in tumor samples and normal samples (Colwill, 2011).

### Somatic mutation and CNA analysis

The mutation data for endometrial carcinoma patients was obtained in “maf” format from the TCGA GDC Data Portal. The R package “maftools” and the “ComplexHeatmap” were used to analyze and visualize the top 20 mutation genes. TMB was defined as the total number of nonsynonymous mutations per megabase in the coding region (Budczies et al., 2018). GISTIC 2.0 and GenePattern (https://www.genepattern.org/) were used to find significant amplifications or deletions in the whole genome for the CNAs. The number of copies greater than one is the threshold for copy amplification, and less than -1 is the threshold for copy deletion.

### Functional and pathway enrichment analyses

We used the R package “clusterprofiler” to perform Gene Ontology (GO) and Kyoto Encyclopedia of Genes and Genomes (KEGG) pathway analyses to functionally annotate cuproptosis-related genes (Yu et al., 2012). Gene Set Variation Analysis (GSVA) was used to investigate the differences between Cu. Score subgroups in biological processes (Hanzelmann et al., 2013). On the other hand, the R package “clusterprofiler” was used to perform Gene Set Enrichment Analysis (GSEA) (Yu et al., 2012). For GSVA and GSEA, the gene sets “h.all.v7.5.1” and “c2. cp.kegg.v7.5.1” were downloaded from the MSigDB database (http://www.gsea-msigdb.org/gsea/index.jsp).

### Estimation of immune infiltration

The ESTIMATE algorithm was used to calculate the immune score, stromal score, ESTIMATE score, and tumor purity (Yoshihara et al., 2013). As previously described, the activity of immune-related pathways in the tumor microenvironment was estimated using single-sample GSEA (ssGSEA) (Tian et al., 2021). Immune cell infiltration was measured using a variety of methods, including ssGSEA, EPIC, TIMER, QUANTISEQ, MCPCOUNTER, XCELL, CIBERSORT, and CIBERSORT-ABS (Newman et al., 2015; Becht et al., 2016; Aran et al., 2017; Finotello et al., 2019; Racle and Gfeller, 2020; Zeng et al., 2021).

### Weighted gene Co-Expression network construction and module identification

WGCNA was performed in this study to screen genes related to cuproptosis using the R package “WGCNA”. The detailed processes were carried out as previously described (Langfelder and Horvath, 2008). We selected genes with the top 25% absolute deviation from the median to screen highly variable genes in the WGCNA expression data. The “goodSampleGenes” function was used to verify the data’s integrity. A standard scale-free network was built using soft threshold power = 4 (scale-free *R* ^2^ = 0.941) in our study. Genes with similar expression profiles were grouped using a dynamic tree-cut algorithm, and similar modules were merged using a height cutoff of 0.5. For further investigation, the module with the highest correlation with cuproptosis genes was chosen. The hub genes were identified at a threshold of the absolute value of gene significance (GS) > 0.20 and the absolute value of module membership (MM) > 0.80.

### Generation of the cuproptosis score (Cu.Score) and cuproptosis-related risk score (Risk.Score)

WGCNA first screened a total of 75 hub genes in the brown module. Based on the expression of 75 hub genes and 13 cuproptosis genes, the ssGSEA algorithm was used to create a cuproptosis score (Cu.Score). The optimal cut-off value obtained by the R package ‘Survminer’ was used to divide the 523 endometrial cancer patients in the entire cohort into high- and low-Cu. Score groups. Also, we constructed a cuproptosis-related risk score (Risk.Score) to find the best biomarker for predicting UCEC prognosis. In simple terms, a univariate Cox regression analysis was first used to perform the prognostic analysis for each gene from the brown module, as well as 13 cuproptosis genes. In the entire cohort with *p* < 0.001, a total of 180 genes with significant prognostic value was extracted for further analysis. In the training cohort, a prognostic signature consisting of 13 genes was established using the least absolute shrinkage and selection operator Cox regression analysis (LASSO-Cox). The Risk. Score was then calculated using the LASSO regression coefficients and 13 prognostic-related gene expression levels. The formula for the Risk. Score was as follows:

$$Risk\;score=\overset{n}{\underset{i=1}{\sum }}Coefi*x_{i}$$

(Coefi stands for coefficients, and $x_{i}$ is the expression level of each prognostic gene) UCEC patients from the training, validation and entire cohorts were divided into low- and high-risk groups based on the training cohort’s median Risk. Score. Its prognostic capability was assessed using a time-dependent receiver operating characteristic (ROC) and a Kaplan-Meier curve analysis. The Risk. Score was verified as an independent prognostic factor in the training, validation, and entire cohorts using univariate and multivariate Cox regression analyses.

### Construction of a predictive nomogram

On the basis of the Risk. Score and clinicopathology factors, the R package “rms” was used to create a nomogram that was used to predict 1-/3-/5-year survival possibilities (overall survival: OS, progression-free survival: PFS). The nomogram’s prognostic value was validated using calibration plots and decision curve analysis. Meanwhile, the concordance index (C-index) was computed to determine the nomogram’s predictive potential.

### Statistical analysis

The log-rank test and Kaplan-Meier were used to examine the statistical significance of differences in the survival analysis. The link between two continuous variables was calculated using Spearman’s correlation coefficient. Student’s t-tests (normally distributed variables) and the Wilcoxon rank-sum test (nonnormally distributed variables) were used to compare a continuous variable between two groups. For comparisons of more than two groups, one-way ANOVA tests and Kruskal–Wallis tests were used as parametric and nonparametric tests, respectively. Chi-square and Fisher’s exact tests were utilized for categorical data. R software was used for all statistical analyses (version 4.0.5). Statistical significance: *, *p* < 0.05; **, *p* < 0.01; ***, *p* < 0.001; ns, not significant.

## Results

### Revealing transcriptional abnormalities and genetic alterations of cuproptosis genes in UCEC

**FIGURE 1:**
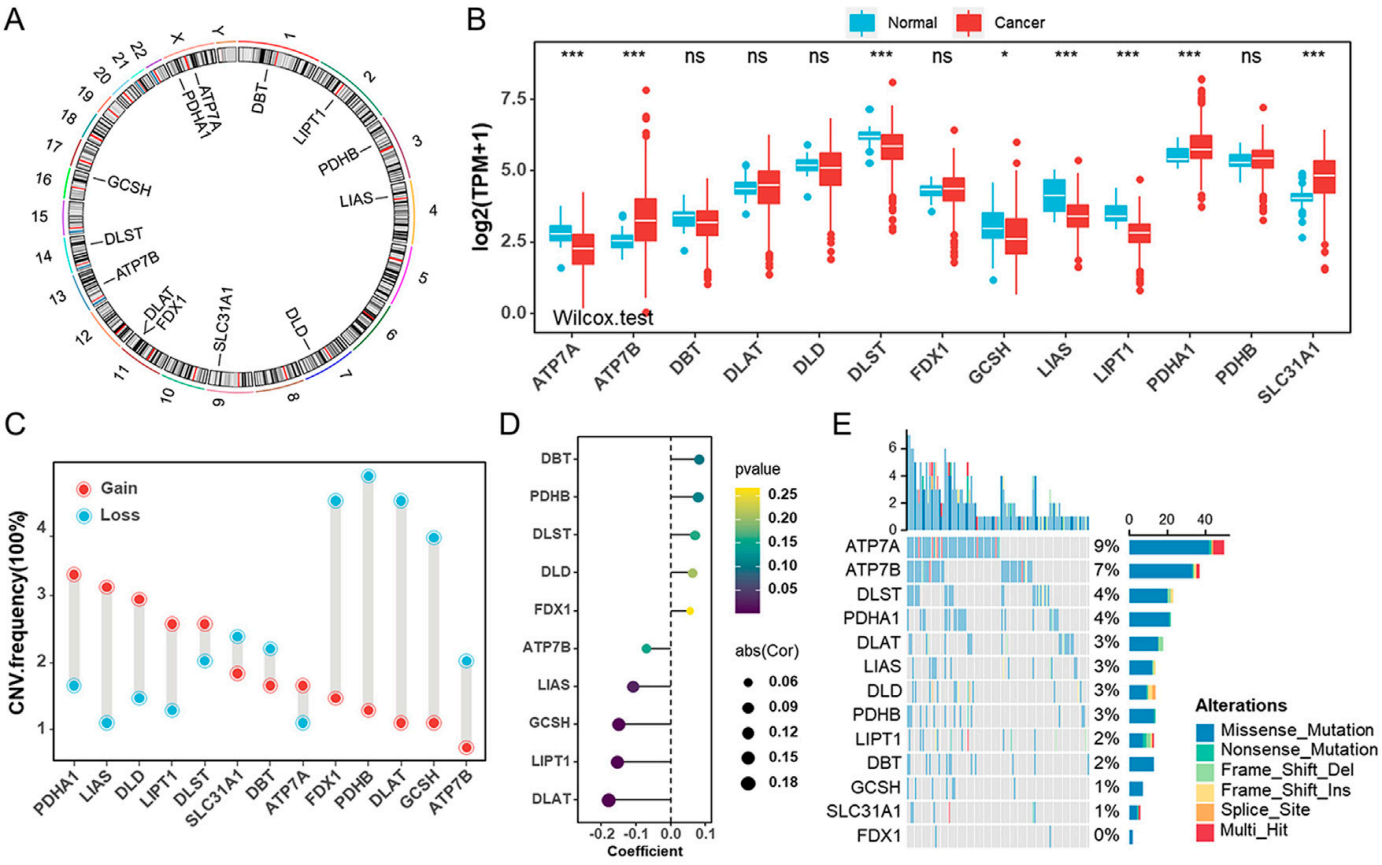
Genetic and transcriptional alterations of cuproptosis genes in endometrial cancer **(A)**. The locations of cuproptosis genes on chromosomes. **(B)** The mRNA levels of 13 cuproptosis genes were compared between normal and tumor tissues in TCGA-UCEC cohort. **(C)** The CNV frequencies of 13 cuproptosis genes in TCGA-UCEC cohort. **(D)** The bubble chart revealing the correlation between the promoter methylation levels of cuproptosis genes and their mRNA levels in TCGA-UCEC cohort. **(E)** The mutation frequencies of 13 cuproptosis genes in TCGA-UCEC cohort.

In this study, we examined the role of 13 cuproptosis genes in UCEC that were identified in recent literature (Tsvetkov et al., 2022). The locations of 13 cuproptosis genes on the chromosomes are shown in Figure 1A. We first compared these genes in endometrial cancer and normal samples from the TCGA-UCEC cohort. The results showed that, in tumor samples, ATP7B, PDHA1, and SLC31A1 were significantly upregulated compared with normal tissues, whereas ATP7A, DLST, GCSH, LIAS, and LIPT1 were significantly downregulated (Figure 1B). Similar results were obtained in the GSE17025 and HPA databases (Supplementary Figure S1A, S1B). To test whether the genetic variation is involved in the dysregulation of cuproptosis gene expression, we examined the frequency change of CNVs in these genes. One of the differentially expressed genes, PDHA1, exhibited widespread copy number variation (CNV) increases, while GCSH experienced CNV decreases (Figures 1B,C). Next, we wondered whether methylation of these genes correlated with corresponding mRNA expression levels. The mRNA levels of DLAT1, LIPT1, and GCSH were negatively correlated with methylation (Figure 1D). Also, we analyzed the incidence of mutations of cuproptosis genes in UCEC. The highest mutation frequency was found for ATP7A (9%), followed by ATP7B (7%) (Figure 1E, Supplementary Figure S1C). Furthermore, UCEC patients with ATP7B mutations tended to have a better prognosis (Supplementary Figure S1D).

### Associating cuproptosis genes with clinical parameters and biological pathways

**FIGURE 2:**
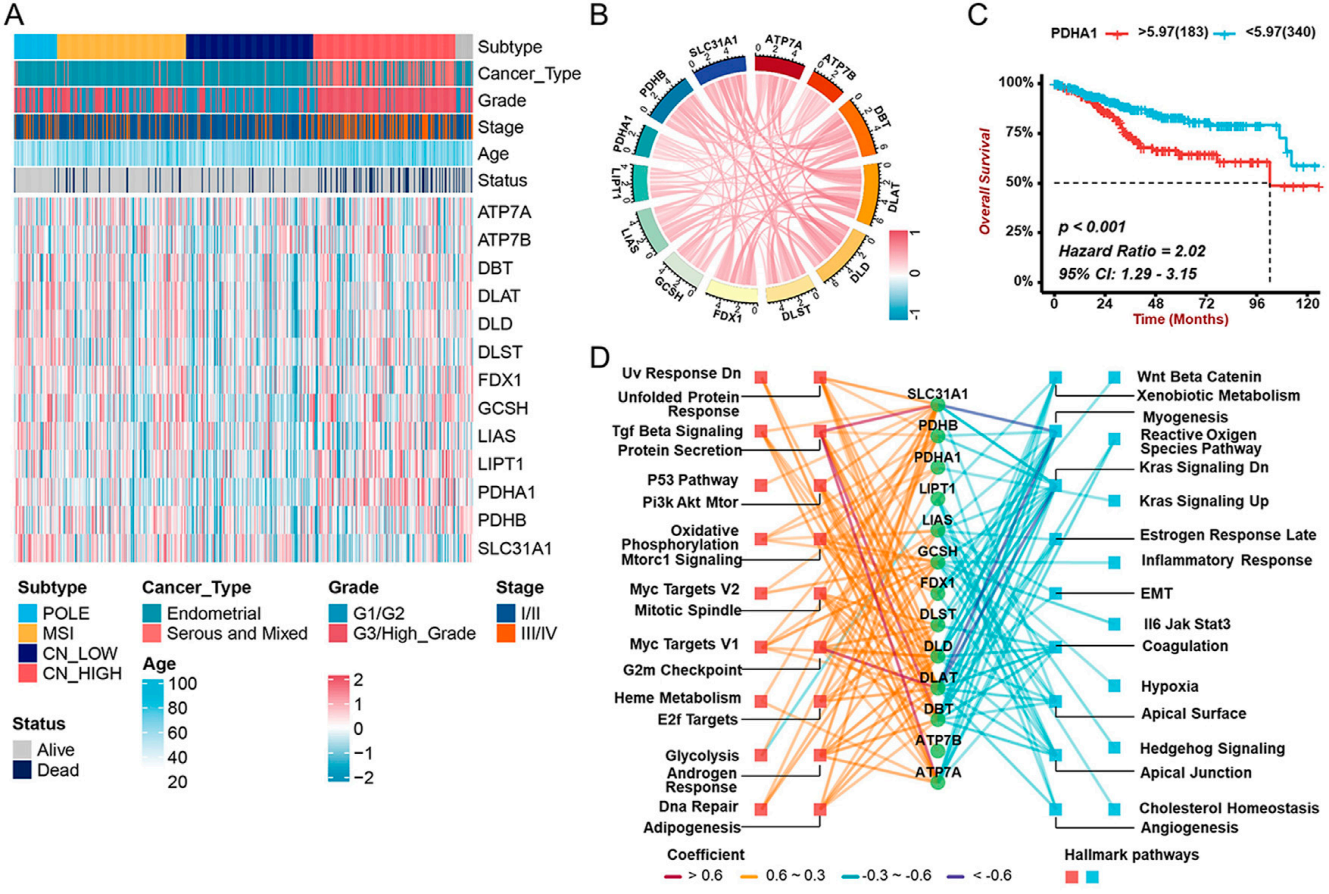
Associations between cuproptosis gene expression and clinicopathological characters and prognostic **(A)** The heatmap revealing the correlations between clinicopathological characters and 13 cuproptosis genes’ expression in TCGA-UCEC cohort. **(B)** The correlation among the mRNA levels of cuproptosis genes in TCGA-UCEC cohort. **(C)** The Kaplan-Meier analysis demonstrating the prognostic significance of PDHA1 in TCGA-UCEC cohort. **(D)** The correlation between cuproptosis genes and cancer hallmark pathways. The colors represent different correlations.

We then examined the correlations between any two cuproptosis genes in the TCGA-UCEC cohort, and the results showed most were positive (Figures 2A,B). A heatmap and box diagram illustrated the correlation between cuproptosis genes and pathological parameters (age, grade, stage, histological type, and TCGA molecular subtype) (Figure 2A, Supplementary Figures S2A–S2E). PDHA1, a subunit of the pyruvate dehydrogenase complex, was significantly increased in individuals with serious histological types or CN-high molecular subtypes. There was significant upregulation of PDHA1 expression among patients with advanced UCEC (elder, older grades and/or stages) and a worse prognosis (Figure 2B, Supplementary Figures S2A–S2E). Other cuproptosis genes like ATP7A, ATP7B, DLD, GCSH, and LIPT1 were highly expressed in patients with poor prognosis, while SLC31A1, with higher expression, was related to prognostic advantages (Supplementary Figure S3A). To study the molecular mechanisms of cuproptosis genes involved in UCEC, we examined the associations between expression of each cuproptosis gene and the status of hallmark pathways. Interestingly, we found that the expression of cuproptosis genes was positively correlated with tumor-related pathways, such as P53, Pi3K-Akt-mTOR, G2m Checkpoint, DNA Repair, and Androgen Response (Figure 2D). Taken together, these findings suggested that cuproptosis genes might play critical roles in the development and progression of UCEC.

### Identifying cuproptosis-related modules and genes by WGCNA in UCEC and annotating their biological functions

**FIGURE 3:**
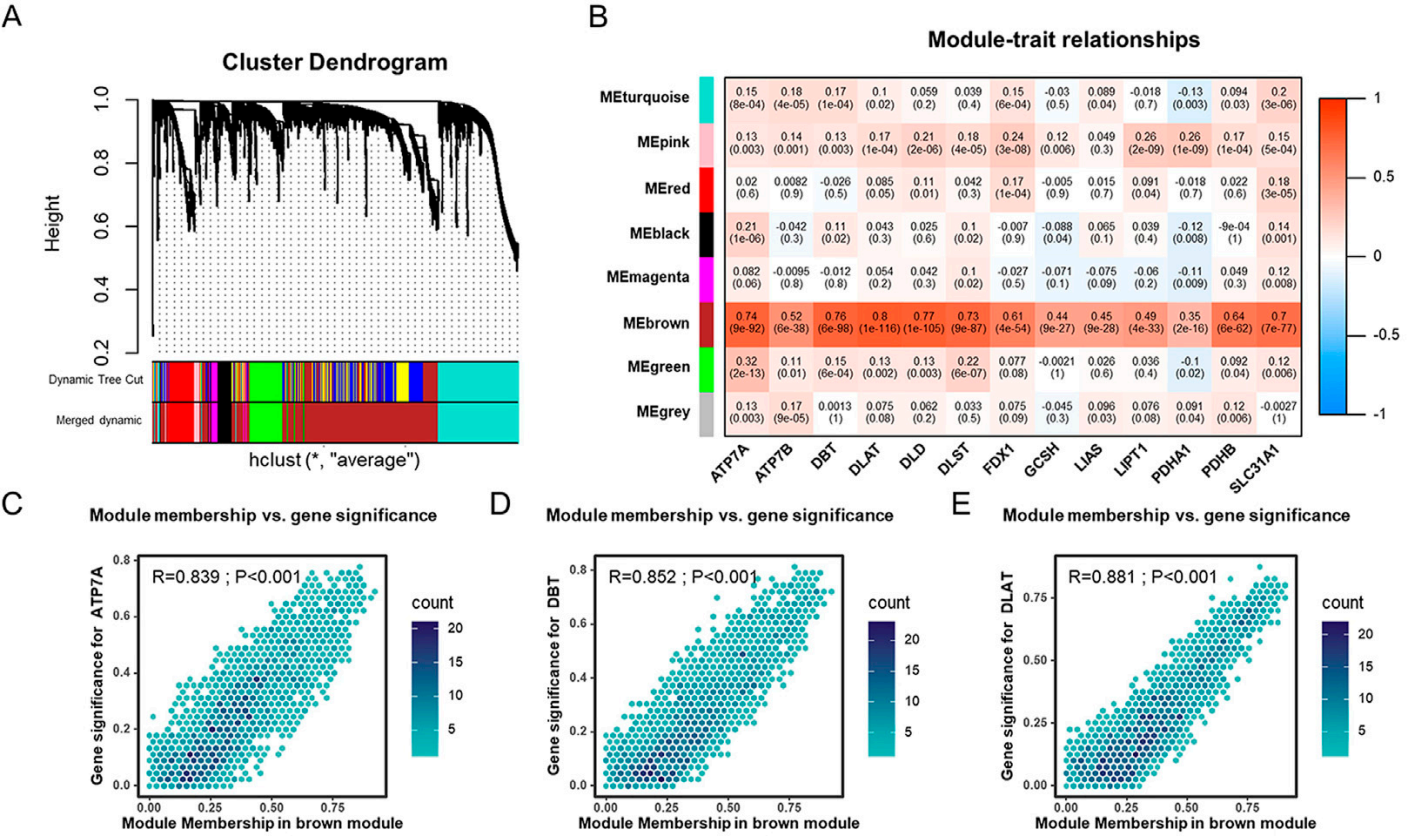
Identification of cuproptosis-related genes in the TCGA-UCEC cohort through WGCNA **(A)**. Dendrogram of all cuproptosis genes clustered based on a dissimilarity measure (1-TOM). **(B)** The heatmap of the correlation between module eigengenes and cuproptosis genes in TCGA-UCEC cohort. **(C–E)** The scatter plots of module eigengenes in MEbrown modules.

As the mechanisms involved in regulating cuproptosis are as yet unclear, we used WGCNA to identify genes related to cuproptosis. Seven modules were identified by WGCNA analysis, among which MEbrown correlated well with cuproptosis genes (Supplementary Figures S3A,B,S4A). MEbrown was significantly correlated with ATP7A, DBT, and DLAT (Figures 3C–E). A total of 2407 genes from MEbrown were considered to be cuproptosis-related genes. To reveal the potential biological functions of those genes, we conducted GO and KEGG analyses (Supplementary Figures S5A, S5B). The GO analysis showed these genes were mostly enriched in functions related to axonogenesis, cell-cell junctions, and growth factor binding (Supplementary Figure S5A). The KEGG pathway enrichment revealed that these genes were mainly associated with axon guidance, cell cycle, and transcriptional misregulation in cancer (Supplementary Figure S5B).

### Constructing the Cu.Score and investigating its clinical and biological relevance

**FIGURE 4:**
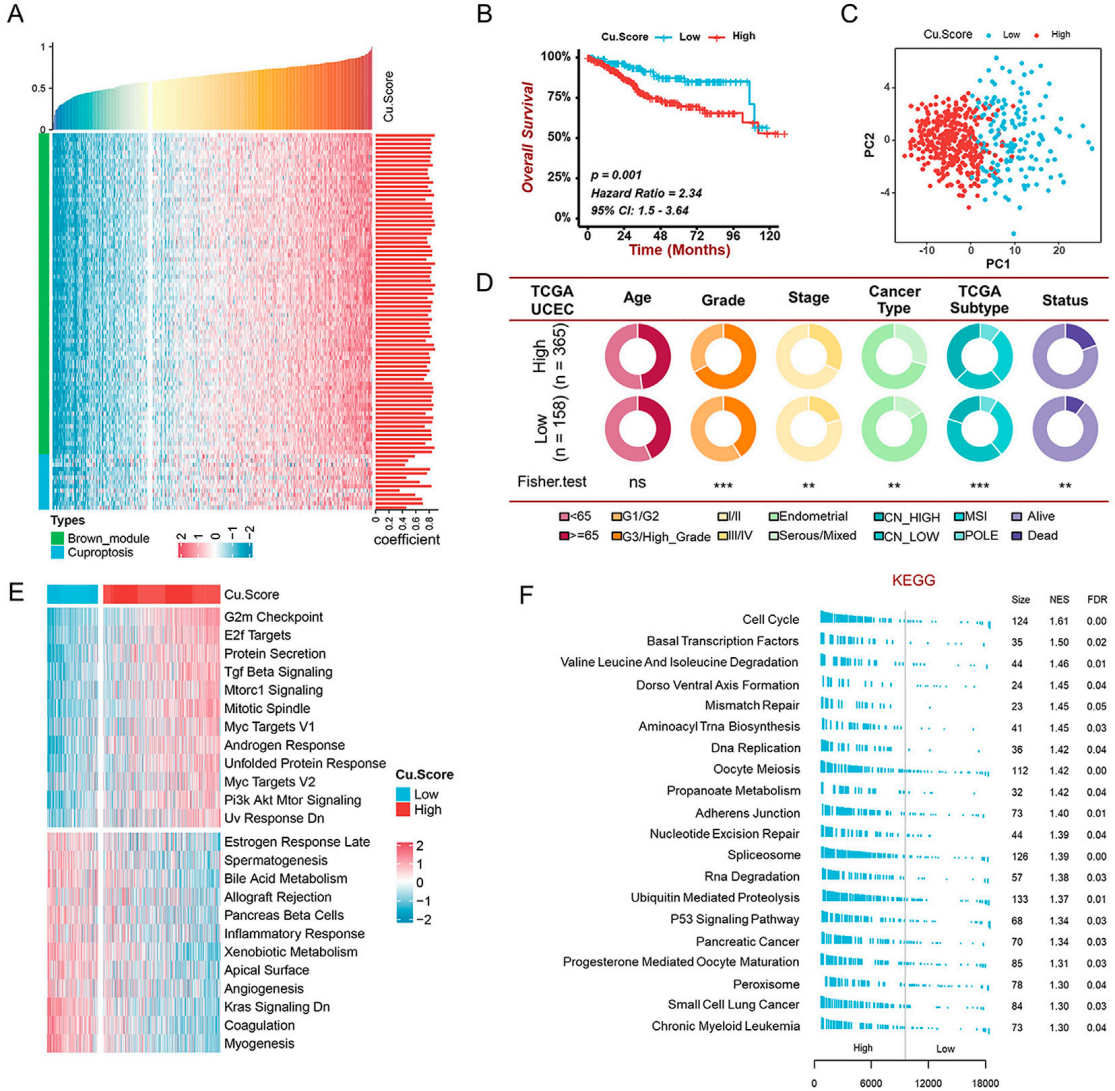
Construction of the Cu. Score and exploration of its clinical significance in the TCGA-UCEC cohort **(A)** The correlation analysis of the relationship between cuproptosis-related genes and Cu. Score. Bar chart on the right indicates the degree of correlation. **(B)** The Kaplan-Meier curve of significant difference in the survival rate between high- and low-Cu. Score groups in TCGA-UCEC cohort. **(C)** The PCA revealing the difference between the high- and low-Cu. Score groups. **(D)** The pie charts showing the Chi-squared test of clinicopathological characters between high- and low-Cu. Score groups. **(E-F)** GSVA **(E)** and GSEA **(F)** showing the status of biological pathways between high- and low-Cu. Score groups.

According to research, hub genes are paramount in managing the behavior of biological modules (Han et al., 2004). We thus identified 75 hub genes in MEbrown with abs (GS) > 0.2 and abs (MM) > 0.8. To assess the level of cuproptosis in patients with UCEC, we constructed a scoring scheme using 75 hub genes and 13 cuproptosis genes (Figure 4A). It was observed that Cu. Score was positively correlated with the above cuproptosis-related genes (Figure 4A). Since cuproptosis genes are related to the clinical characteristics and prognosis of UCEC patients, the UCEC patients were classified into high- and low-Cu. Score groups based on the optimal cut-off value. Kaplan-Meier survival curves revealed that patients with a lower Cu. Score had better clinical outcomes (Figure 4B). The principal component analysis (PCA) demonstrated that the expressions of hub genes and cuproptosis genes could distinguish the two groups well (Figure 4C). Moreover, the high-Cu. Score group showed poorer disease progression and survival status than the low-Cu. Score group (Figure 4D). Next, we performed GSVA and GSEA to investigate biological molecular changes between two Cu. Score groups (Figures 4E,F). On one hand, pathways associated with tumorigenesis, like G2M checkpoints, E2F targets, TGF-beta signaling, MYC targets, and PI3K-AKT-mTOR, are primarily enriched in the high-Cu. Score group. On the other hand, immune-related pathways, like the inflammatory response, are mainly enriched in the low-Cu. Score group (Figures 4E,F). This suggests that the high-Cu. Score groups were related to tumor-related pathways, whereas the low-Cu. Score groups were related to immune-related pathways.

### Characteristics of the tumor immune microenvironment in different Cu.Score subgroups

**FIGURE 5:**
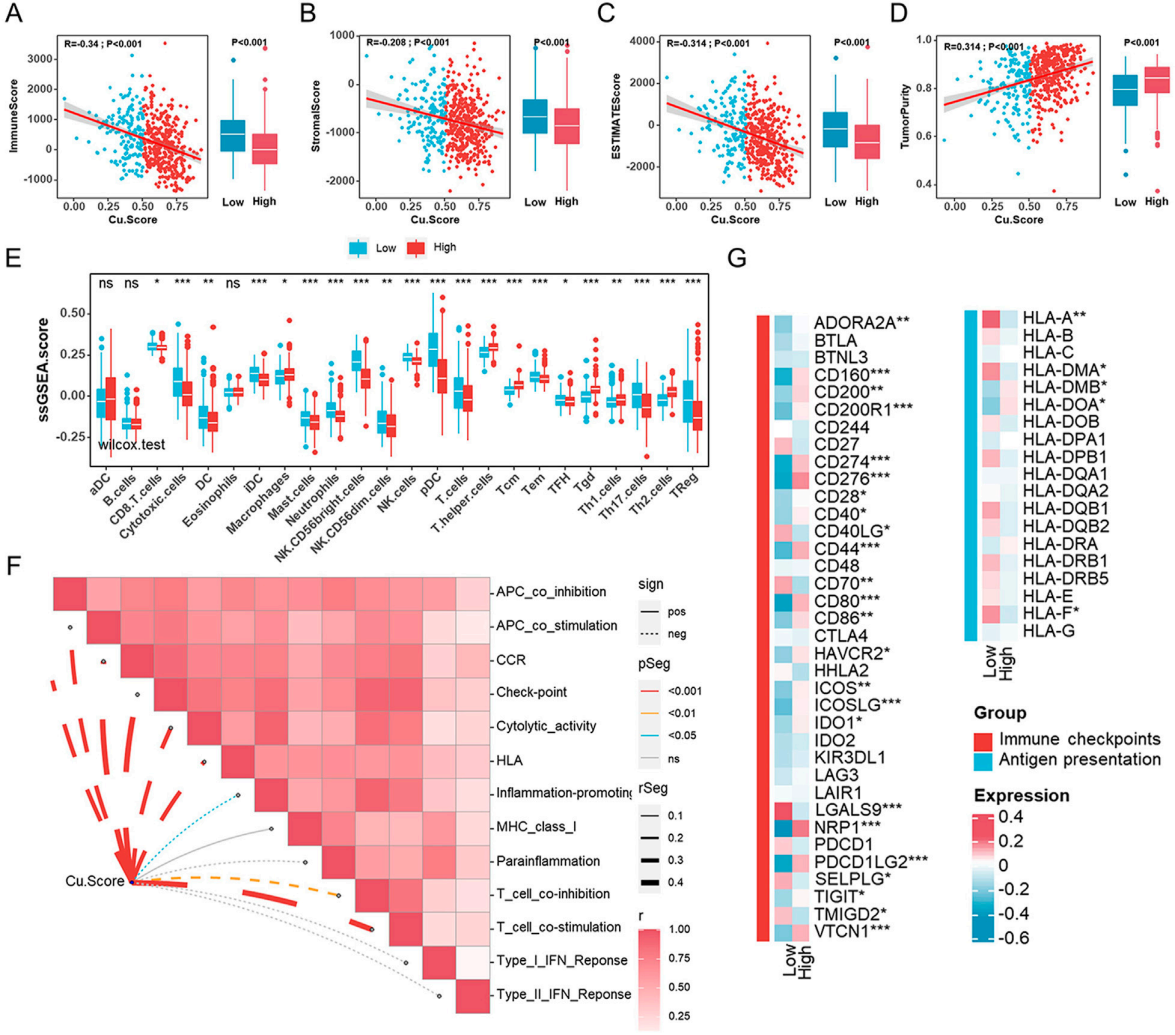
Characteristics of the tumor microenvironment in different Cu. Score subgroups **(A–D)**. The correlation analyses between Cu. Score with ImmuneScore **(A)**, StromalScore **(B)**, ESTIMATEScore **(C)**, and TumorPurity **(D)**. **(E)** Comparisons of the abundances of 24 immune cells in two subgroups. **(F)**. The relationships between Cu. Score and immune-related function scores. **(G)** The heatmap of the comparison of the immune checkpoints and HLA genes between the high- and low-Cu. Score groups.

The effect of cuproptosis on the TME of UCEC was investigated using ESTIMATION analysis to compare immune status between Cu. Score subgroups (Figures 5A–D). ImmuneScore, StromalScore, and ESTIMATEScore were significantly lower in people with a higher Cu. Score. Patients with a high Cu. Score had higher TumorPurity than those with a lower Cu. Score (Figures 5A–D). Additionally, we carried out a correlation analysis to determine the relationships between the two subgroups and immune cells and immune-related functions using the ssGSEA algorithm. Infiltration levels of CD8^+^ T cells, cytotoxic cells, DC, iDC, macrophages, mast cells, neutrophils, NK CD56 (bright) cells, NK cells, pDC, Th17, and Tregs cells were higher in the low-Cu. Score group compared to the high-Cu. Score group, but T helper cells, Tcm, Tgd, Th1 cells, and Th2 cells were significantly lower in the low-Cu. Score group (Figure 5E). Consistently, most immune cells were significantly higher in the low-Cu. Score group, which was confirmed by cross-validation with EPIC, TIMER, QUANTISEQ, MCPCOUNTER, XCELL, CIBERSORT, and CIBERSORT-ABS (Supplementary Figure S6A). We further examined the associations between the expression of 13 cuproptosis genes and the tumor-infiltrating immune cells. The expression of cuproptosis genes was positively correlated with T-helper cells, Tcm, Tgd, and Th2 cells, as well as negatively correlated with other immune cells (Supplementary Figure S7A). Meanwhile, the Cu. Score was negatively related to most immune-related functions and cancer immunity cycles (Figures 5F, Supplementary Figure S7B). Moreover, we examined the differences between the high- and low-Cu. Score groups in terms of immune checkpoints and HLA genes. Only a few immune checkpoints and HLA genes were expressed differently between the two subgroups (Figure 5G).

### Discovering genetic alterations and estimating drug sensitivity in high- and Low-Cu.Score subgroups

**FIGURE 6:**
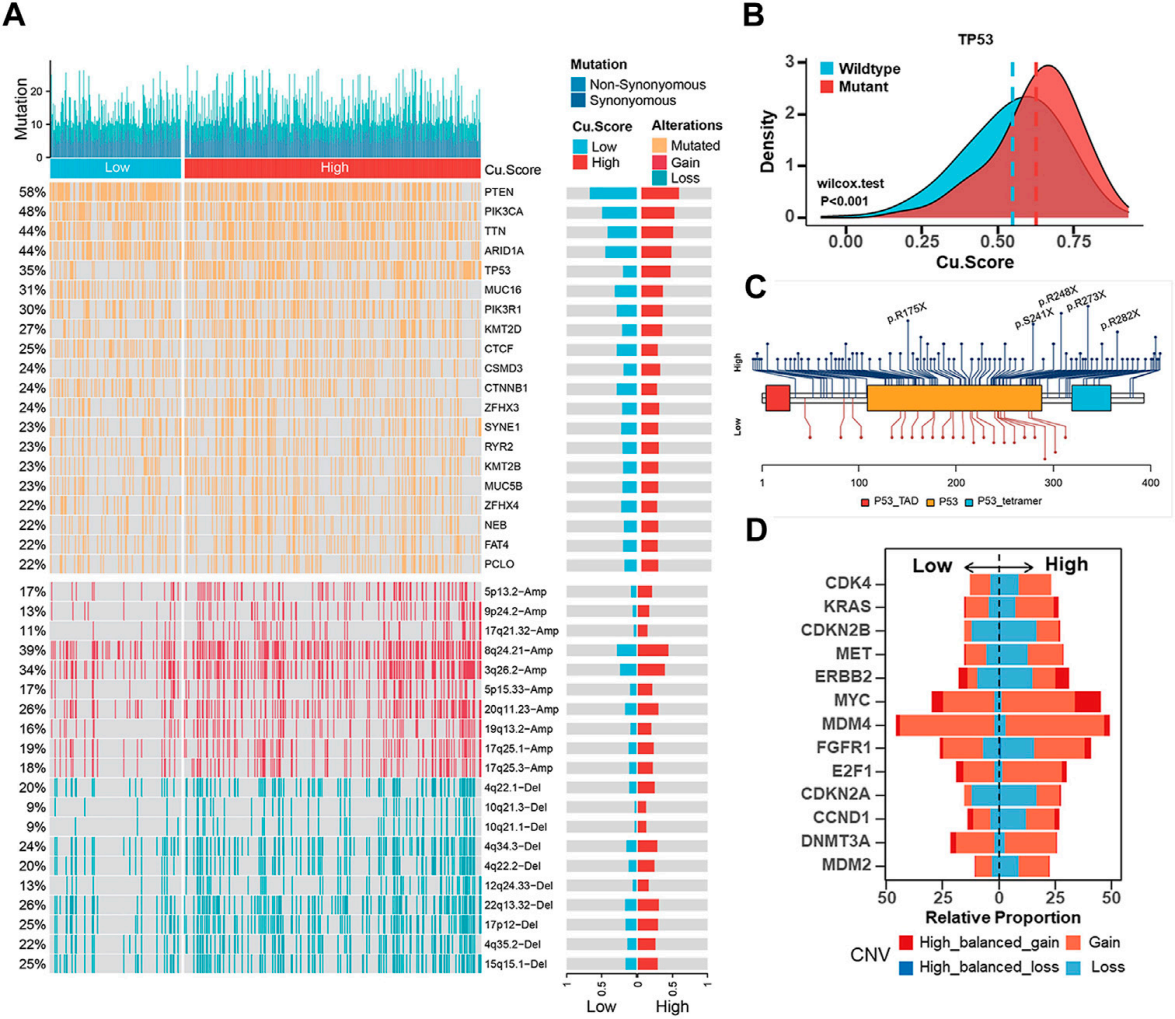
Estimation of genetic alterations in high- and low-Cu. Score subgroups **(A)**. Comparisons of somatic mutations (The top half) and CNAs (The bottom half) between different Cu. Score subgroups. **(B)** The difference of Cu. Score between the TP53 mutant group and the TP53 wild-type group. **(C)** The lollipop plot of the differential distribution of variants for TP53 in TCGA-UCEC cohort. **(D)** The CNV frequencies of some representative oncogenes or tumor suppressor genes in TCGA-UCEC cohort.

In tumorigenesis, genetic alterations like somatic mutations and CNAs play significant roles. We examined somatic mutations and CNVs to reveal differences between the high- and low-Cu. Score groups at the genomic level in the TCGA-UCEC cohort. The high-Cu. Score group presented more synonymous mutations than the low-Cu. Score group (Supplementary Figures S8A, S8B). A further analysis was performed on the top 20 mutated genes. TP53 mutations were more frequent in high-Cu. Score groups, while PTEN and CTNNB mutations were prominent in low-Cu. Score groups (Figure 6A). Further analysis revealed that those with TP53 mutations achieved a higher Cu. Score (Figure 6B). A lollipop plot revealed the differences in mutation spots of TP53 between the two subgroups (Figure 6C). The TP53 mutation is considered a surrogate biomarker of the serous-like “copy number high” UCEC subtype (Singh et al., 2020). As shown in Figure 6A, the Cu. Score group had more CNV-altered regions. In detail, several oncogenes or tumor suppressor genes were amplified or deleted, respectively, in the high-Cu. Score group, including MYC, FGFR1, ERBB2 (HER2), E2F1, CDKN2A, CDKN2B, DNMT3A, MET, CCND1, and CDK4 (Figure 6D). According to prior studies, patients with high TMB and MSI-L/H have a better prognosis (Liu et al., 2021; Liu et al., 2022). Thus, we examined the effect of TMB/MSI combined with Cu. Score on the prognosis of UCEC patients. The results showed that TMB/MSI combined with Cu. Score could better predict the survival of UCEC patients (Supplementary Figures S8C, S8D). What’s more, we analyzed correlations between the Cu. Score and IC_50_ of drug candidates in the GDSC database and selected six chemotherapeutic agents to evaluate the IC_50_ of these drugs in the high- and low-Cu. Score groups. The patients in the high-Cu. Score group had lower IC_50_ values for cisplatin, docetaxel, doxorubicin, and gemcitabine, while the IC_50_ values of chemotherapeutics such as imatinib and lapatinib were significantly lower in the patients with low Cu. Score (Supplementary Figure S9A).

### Constructing and validating the cuproptosis-related prognostic signature

**FIGURE 7:**
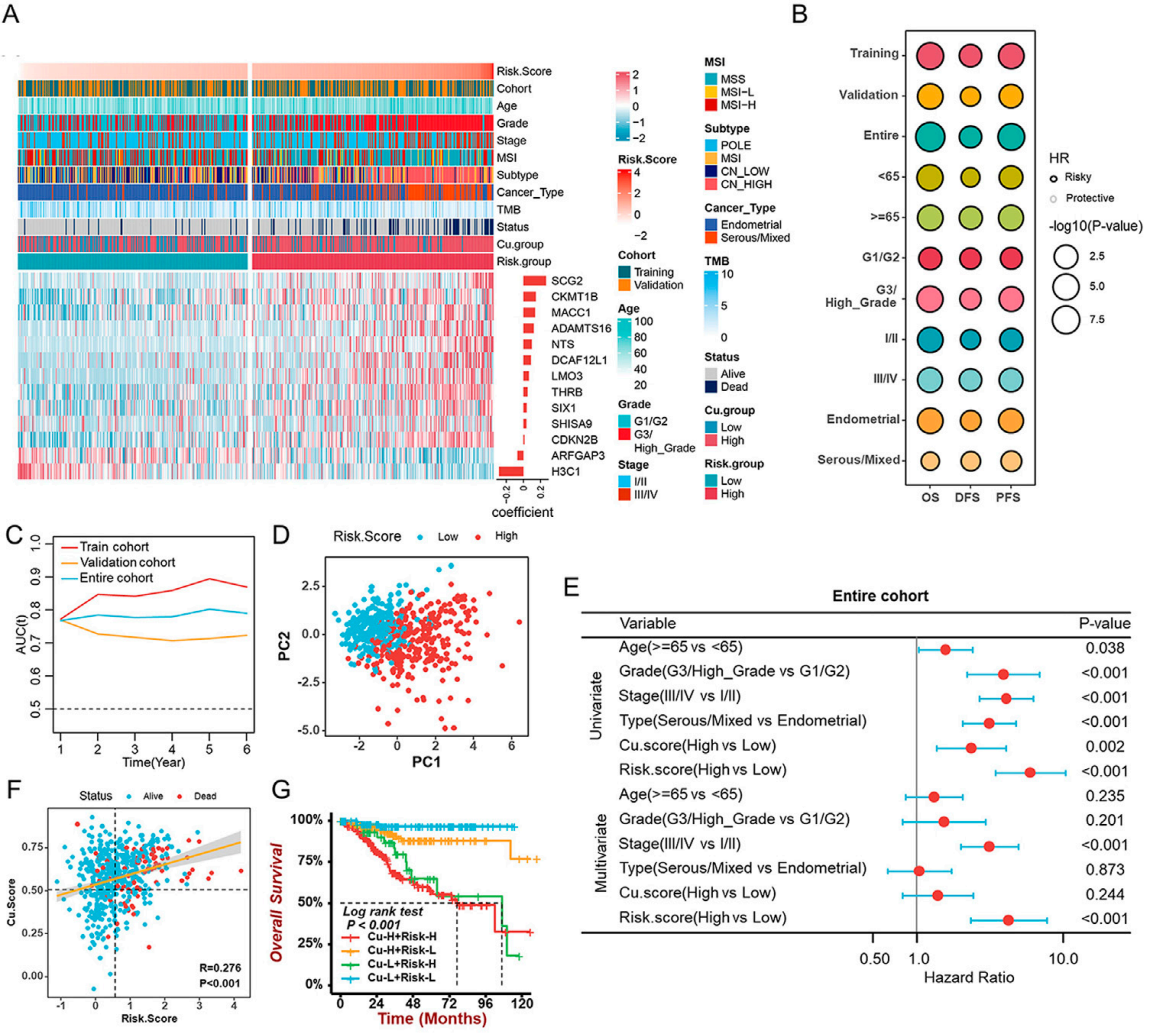
Construction and validation of the cuproptosis-related prognostic signature **(A)**. The heatmap revealing the correlations between clinicopathological characters and 13 cuproptosis-related prognostic genes **(B)**. The prognostic performance of the cuproptosis-related prognostic signature in different cohorts, age, grade, stage, and histological types. **(C)** The time-dependent AUC value in the training, validation, and entire cohorts. **(D)** The PCA revealing the difference between the high- and low-risk groups based on 13 cuproptosis-related genes. **(E)**. The univariate and multivariate Cox analyses suggesting the independent prognostic value of the Risk. Score in the entire cohort. **(F)** The correlation between Cu. Score and Risk. Score. **(G)** The survival analyses of patients with different Cu. Score combined with the Risk. Score.

To identify the best biomarker for the prognosis of UCEC based on cuproptosis, we randomly divided the entire cohort (523 tumor samples) into the training cohort (*n* = 262) and the validation cohort (*n* = 261) in a 1:1 ratio (Supplementary Figure S10A). In the entire cohort, we discovered 180 cuproptosis-related genes that were associated with survival using univariate Cox regression. To generate a cuproptosis-related prognostic signature model, LASSO-Cox regression analysis was applied next in the training cohort, and 13 genes from MEbrown were selected for further study (Figure 7A, Supplementary Figures S10B,S10C). In this case, we established a prognostic signature correlated with cuproptosis and calculated the Risk. Score as follows:

Risk.Score = 0.121 * ADAMTS16–0.07 * ARFGAP3 + 0.012 * CDKN2B + 0.145 * CKMT1B + 0.088 * DCAF12L1 -0.285 *H3C1 + 0.064 * LMO3 + 0.137 * MACC1 + 0.098 * NTS + 0.264 * SCG2 + 0.036 * SHISA9 + 0.039 * SIX1 + 0.049 * THRB.

In the training cohort, the median Risk. Score was used to separate samples into high- and low-risk groups. The Kaplan-Meier survival analysis and the log-rank test revealed the survival rate of patients with low Risk. Score was higher than that of patients with a high score (Figure 7B). Further analysis of the 13 genes’ prognostic values was conducted on the validation cohort and the entire cohort (Figure 7B). In addition, the Risk. Score achieved satisfactory prognostic discrimination in patients with age, grade, stage, and histological type (Figure 7B). Meanwhile, the time-dependent Area Under Curve (AUC) suggested the Risk. Score had a significant effect on predicting the prognosis of UCEC, no matter in the training, validation, or entire cohorts (Figure 7C). PCA showed a discrepancy between the high- and low-risk groups (Figure 7D). Subsequently, combined with both univariate and multivariate Cox regression analyses, the Risk. Score can be considered an independent prognostic factor for UCEC (Figure 7E, Supplementary Figures S10D, S10E). Importantly, to further evaluate the predictive performance of the risk score in UCEC patients, we compared the Risk. Score, Cu. Score, the Wang’s sig, and the Yao’s sig (Yang et al., 2021; Chen et al., 2022) and discovered that the AUC of OS for the Risk. Score is 0.753, which is significantly higher than that of other signatures (Supplementary Figure S10F). Further investigation suggested a positive correlation between Risk. Score and Cu. Score (Figure 7F). A survival analysis of Risk. Score combined with Cu. score indicated that patients with high Risk. Score as well as Cu. Score had a lower survival rate than other patients (Figure 7G). Additionally, we discovered that the TMB/MSI and Risk. Score together could more accurately predict the survival of UCEC patients (Supplementary Figures S10G, S10H).

### Establishing a nomogram for predicting survival

**FIGURE 8:**
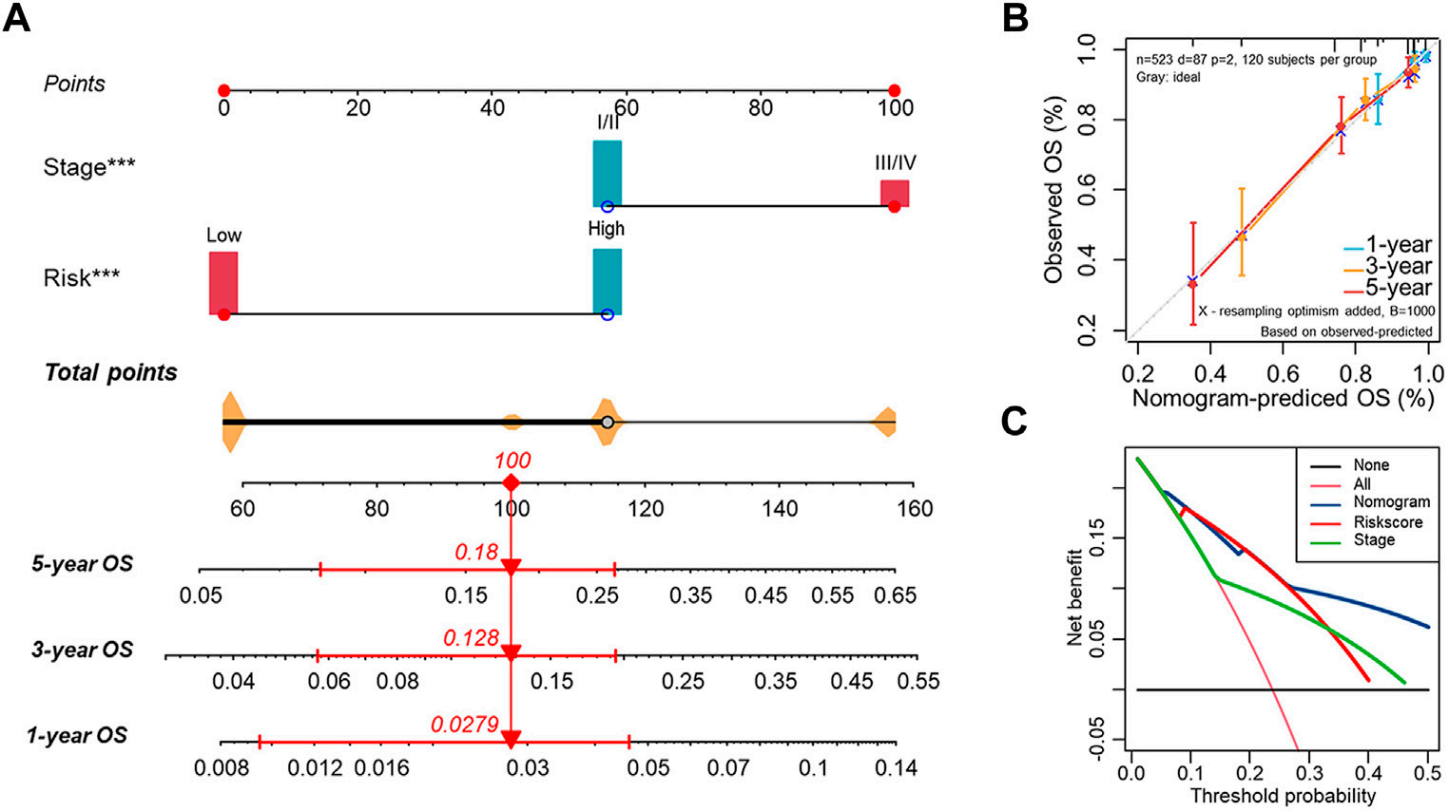
Establishment of the nomogram for predicting OS of endometrial cancer patients in the entire cohort **(A)** The nomogram predicting the probability of the 1-/3-/5-year overall survival rate of UCEC patients. **(B)** The calibration curve for evaluating the accuracy of the nomogram model. The dashed diagonal line in grey color represents the ideal nomogram. **(C)** The decision curve analysis showing predicted 5-year OS based on the nomogram, the Risk. Score, and the stage.

Based on the Risk. Score and clinical factors, we further constructed a nomogram that incorporated the Risk. Score into the entire set to forecast the probability of survival of UCEC patients within certain periods (Figure 8A). Each UCEC patient can obtain a score calculated based on their prognostic parameters to predict their 1-/3-/5-year survival probability (OS, PFS) (Figure 8A, Supplementary Figure S11A). The higher the overall score, the worse the outcome. Nomogram prediction and actual observation reached an excellent agreement on the 1-/3–5-year survival probability after calibration (Figure 8B, Supplementary Figure S11B). Moreover, the Decision Curve Analysis (DCA) revealed that our nomogram had greater net benefits in terms of survival than other parameters (Figure 8C, Supplementary Figure S11C). It was also noteworthy that the C-index showed that the nomogram had a consistent and robust ability to predict outcomes across the entire cohort (OS: C-index = 0.755; PFS: C-index = 0.687) (Supplementary Figure S13D).

## Discussion

The cuproptosis process is an alternative mode of cell death to known apoptosis, ferroptosis, necroptosis, and iron death (Tsvetkov et al., 2022). The role of cuproptosis, especially in tumorigenesis, remains unclear. Our research focuses on the relationship between the cuproptosis gene and endometrial cancer. We discovered significant differences in the expression levels of the majority of cuproptosis genes between tumor and normal samples. Further analysis of the relationship among mRNA expression, CNAs, and DNA methylation revealed that the up-regulation of PDHA1 expression might be related to an increase in copy number, and abnormal expression of LIPT1 might be due to DNA methylation. Moreover, the cuproptosis genes were discovered to be strongly related to pathological parameters and the prognosis of UCEC.

Copper is an essential micronutrient, and its deficiency affects many important cellular functions. However, excess copper is toxic as well (Kuo et al., 2012). The equilibrium level of cellular Cu is maintained by a balance among the Cu transporters (CTR1, also known as SLC31A1), Cu storage (CTR2), Cu chaperones (ATOX1 for intracellular distribution), and Cu exporters (ATP7A and ATP7B) (Ferenci, 2006; Kuo et al., 2012). Overexpression of ATP7B in endometrial carcinoma was correlated with poor outcomes in patients treated with cisplatin-based chemotherapy (Aida et al., 2005). The ATP7B gene mutation, resulting in copper overload and autistic copper homeostasis, is the cause of Wilson’s disease (Czlonkowska et al., 2018). Our study revealed that ATP7B was up-regulated in tumor tissues, and patients who expressed high levels of ATP7B had a poor prognosis. Compared to patients with wild-type ATP7B, those with mutant ATP7B had a better prognosis. In patients with POLE or MSI subtypes, SLC31A1 expression is often overexpressed, and the prognosis of patients improved when its expression was elevated. According to Tsvetkov et al., copper-dependent death is caused by copper binding directly to lipoylated components of the TCA. Eventually, lipoylated proteins aggregate, and iron-sulfur cluster proteins are lost, which results in proteotoxic stress and cell death (Tsvetkov et al., 2022). In addition, SLC31A1 overexpression dramatically increased sensitivity to physiological copper concentrations (Tsvetkov et al., 2022). Pyruvate dehydrogenase complexes are rate-limiting enzyme complexes that help maintain the TCA cycle. These enzyme complexes convert pyruvate to Ac-CoA and link glycolysis with oxidative phosphorylation (Sun et al., 2015). A recent study suggested that PDHA1, a subunit of the PDH complex, helped breast cancer cells adapt to metabolic and oxidative stresses (Cai et al., 2020). Jingjing Chen et al. also found that PDHA1 controlled lipid biosynthesis during prostate cancer progression (Chen et al., 2018). Our study had shown that individuals with advanced UCEC (elderly, higher grades, and/or stages) and worse prognosis had significantly higher expression of PDHA1.

Not limited to a few cuproptosis genes, our study used WGCNA to explore many potential cuproptosis-related genes. We identified a total of MEbrown and 75 hub genes relevant to cuproptosis. Based on the 75 hub genes and 13 cuproptosis genes, we constructed a scoring system termed the Cu. Score. A higher Cu. Score tended to represent a worse prognosis. In addition, carcinogenic pathways were activated in the high-Cu. Score group in enrichment analyses based on GSVA and GSEA, whereas the low-Cu. Score groups were linked to immune regulation and stromal-related signaling.

There is increasing evidence supporting the importance of TME for cancer progression and therapeutic response (Zeng et al., 2019). By using ESTIMATE to analyze the relationship between Cu. Score and immune status, we discovered that low-Cu. Score groups had higher ImmuneScores, StromalScores, and ESTIMATEScores. The TME context correlated with the immune response and benefit of chemotherapy, and different dominant cell populations within the TME could result in distinct clinical outcomes of cancers (Zeng et al., 2019). CD8^+^ T cells, cytotoxic cells, DC, iDC, macrophages, Mast cells, neutrophils, NK CD56bright cells, NK cells, pDC, Th17, and Treg cells were predominant in samples with a low Cu. Score. Patients with a high Cu. Score had higher infiltration of T helper cells, Tcm, Tgd, Th1 cells, and Th2 cells. There were also some correlations between tumor-infiltrating cells and cuproptosis genes. A negative correlation was also found between the Cu. Score and the activities of several immune-related functions and cancer immunity cycles. Treg cells, which suppress aberrant immune responses against self-antigens, also suppress anti-tumor immune responses. Infiltration of lots of Treg cells into tumor tissues is often associated with a poor prognosis (Tanaka and Sakaguchi, 2017). In our study, the low Cu. Score group contained various immune cells, but their functions may be suppressed by immune regulatory cells. Recent studies have shown that immunosuppressive blockers such as the inhibitors of programmed death ligand 1 (PD-L1) and cytotoxic T-lymphocyte-associated protein-4 (CTLA-4) may be used as novel treatment targets (Powles et al., 2020). There was no significant difference between the two Cu. Score groups in the expression level of immune checkpoints. TMB represents the total number of coding mutations in a tumor and is related to the emergence of neoantigens that trigger antitumor immunity (Allgauer et al., 2018). Studies have shown that TMB is a valuable biomarker to predict patient response to PD-L1 treatment (Topalian et al., 2016). The Cu. Score and TMB only exhibited a weak correlation. Based on our findings, a low Cu. Score was characterized by the presence of immune cells and immune regulatory cells, as well as patterns of angiogenesis, which corresponded to the immune excluded phenotype, whereas a high Cu. Score was characterized by an immunosuppressive TME, resembling the immune desert phenotype.

The evaluation of CNVs and somatic mutations at the genome level is fundamental to cancer diagnosis and treatment. Our findings revealed that the TP53 mutation rate and CNV alteration frequency were elevated in the high-Cu. Score subgroup. The high-Cu. Score subgroup exhibited p53 mutations and HER2 amplification, while the low-Cu. Score subgroup exhibited mutations in PTEN, PIP3CA, ARID1A, and CTNNB1. Further analysis was carried out on the relationship between Cu. Score and chemotherapy. Those in the high-Cu. Score group might be more sensitive to cisplatin, docetaxel, and doxorubicin, while those in the low-Cu. Score group could benefit from imatinib and lapatinib. As for individualized therapy, selecting potentially sensitive agents for UCEC patients according to the Cu. Score may improve clinical outcomes. These agents can be used as complementary agents in combination therapy or as new options for the treatment of first-line drug resistance.

The Cu. Score, constructed with 75 hub genes in MEbrown and 13 cuproptosis genes, reveals the cuproptosis levels of patient samples. To identify the best biomarker to predict the prognosis of UCEC patients, we also developed a risk prediction model signature of 13 cuproptosis-related prognostic genes and classified UCEC patients into high- and low-risk groups. In the log-rank test, ROC curve analyses, univariate and multivariate Cox regression analyses, our signature exhibited great capability in predicting OS or PFS, suggesting that the Risk. Score is a reliable prognostic indicator for UCEC patients. A nomogram using a combination of the risk scores and stage was found to be more effective than other clinical features.

There are still some limitations to our study. Firstly, the data is mostly from TCGA, with a single data source and a small sample size, which requires verification across multiple datasets. Moreover, the cuproptosis-related modules and genes discovered through WGCNA still need to be further validated through experimental and clinical studies. In addition, to fully comprehend the clinical significance of cuproptosis, more prognosis-related factors should be contained and analyzed.

## Conclusion

To conclude, we conducted a comprehensive analysis of cuproptosis-related genes and developed a cuproptosis scoring system and a prognostic prediction model for UCEC. The clinicopathological features, enriched pathways, components in the immune microenvironment of UCEC, genomic alterations, and chemotherapy selection were widely investigated in two different Cu. Score groups. Furthermore, the Risk. Score was confirmed to be an independent prognosis factor of UCEC and was included to construct a nomogram. The findings might help to improve our understanding of cuproptosis in tumors and provide new ideas for treating UCEC patients individually.

## Data Availability

The datasets presented in this study can be found in online repositories. The names of the repository/repositories and accession number(s) can be found in the article/Supplementary Material.
