# Supplementary figures and images for: A comprehensive analysis focusing on cuproptosis to investigate its clinical and biological relevance in uterine corpus endometrial carcinoma and its potential in indicating prognosis
Source: Front Mol Biosci. 2022 Dec 7;9:1048356. doi: 10.3389/fmolb.2022.1048356 (PMC9767979; doi:10.3389/fmolb.2022.1048356)

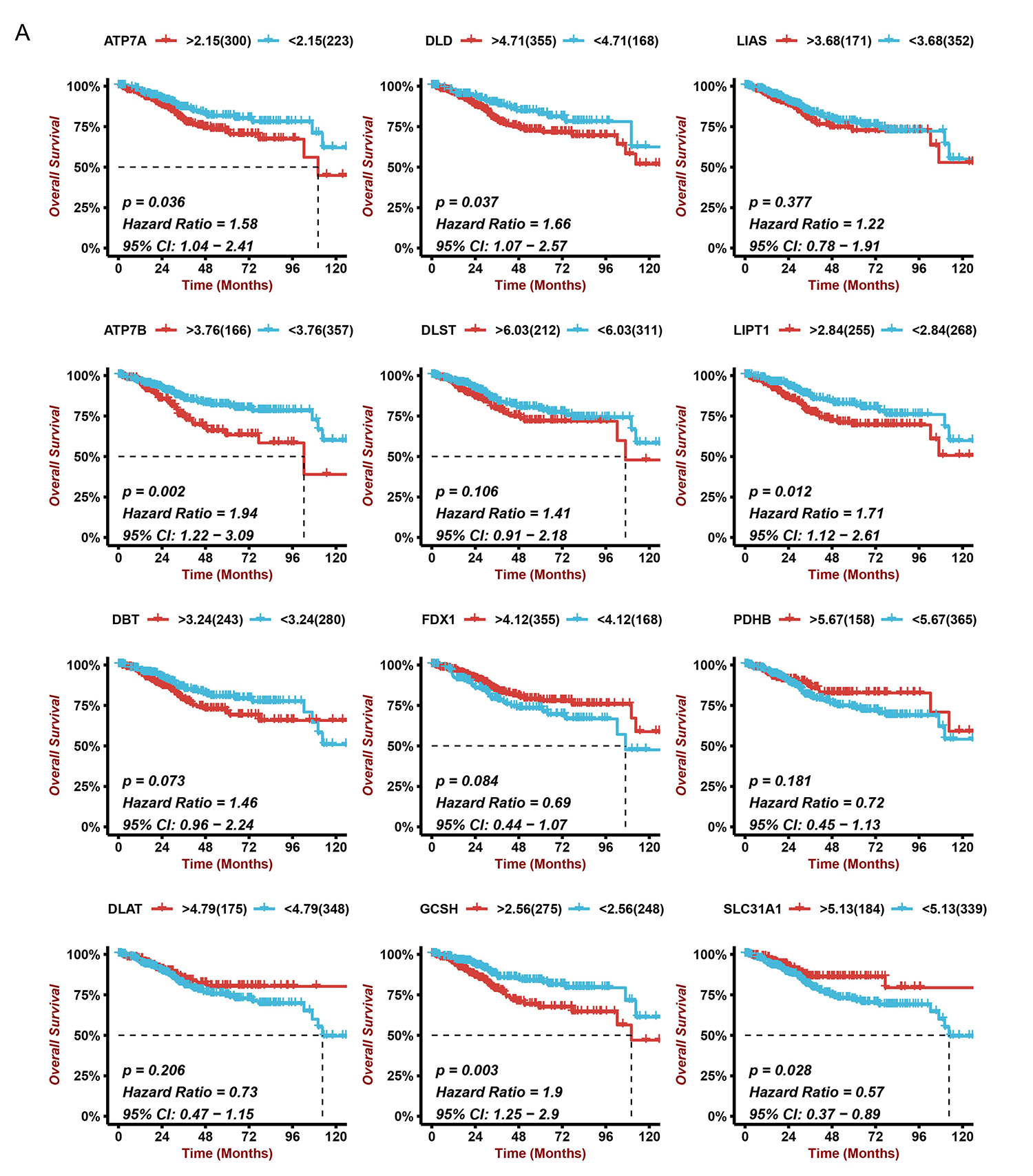

Supplement: Supplementary file 1 [file Image3.JPEG]

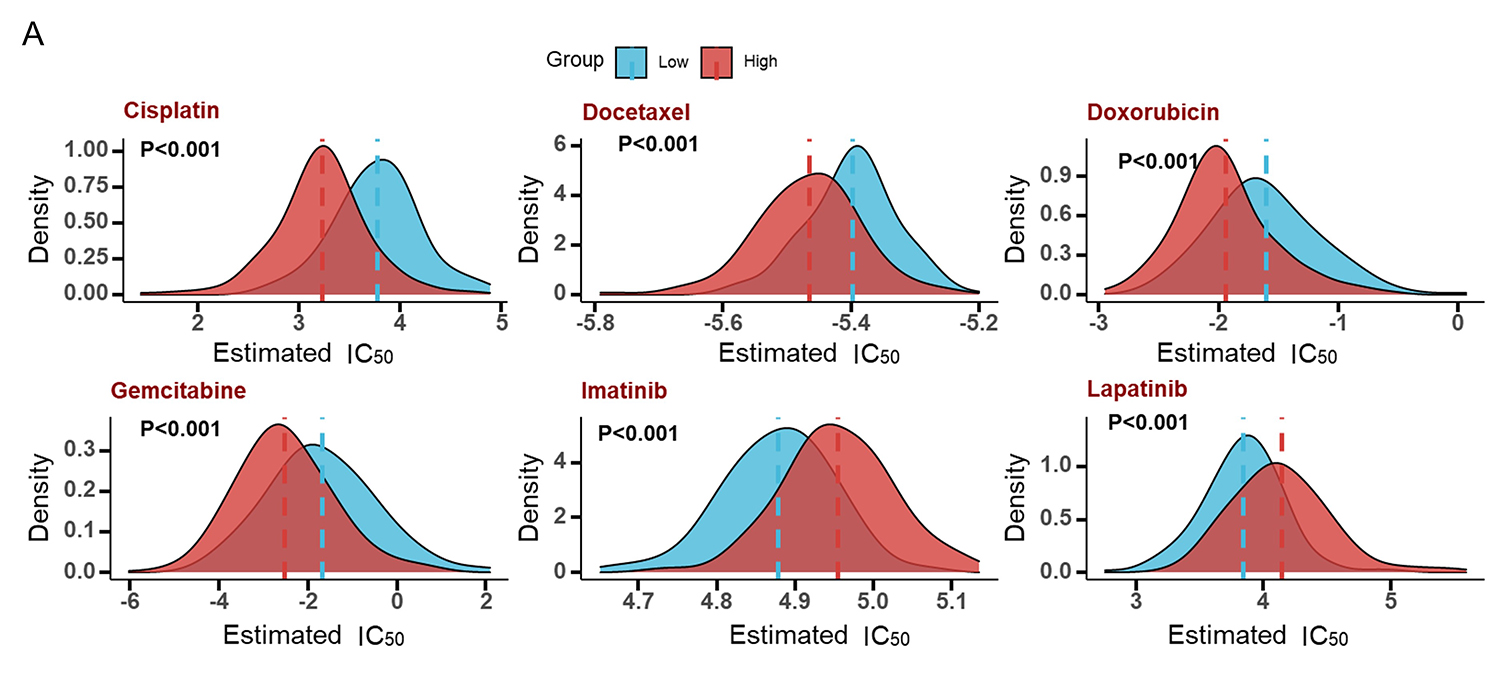

Supplement: Supplementary file 2 [file Image9.JPEG]

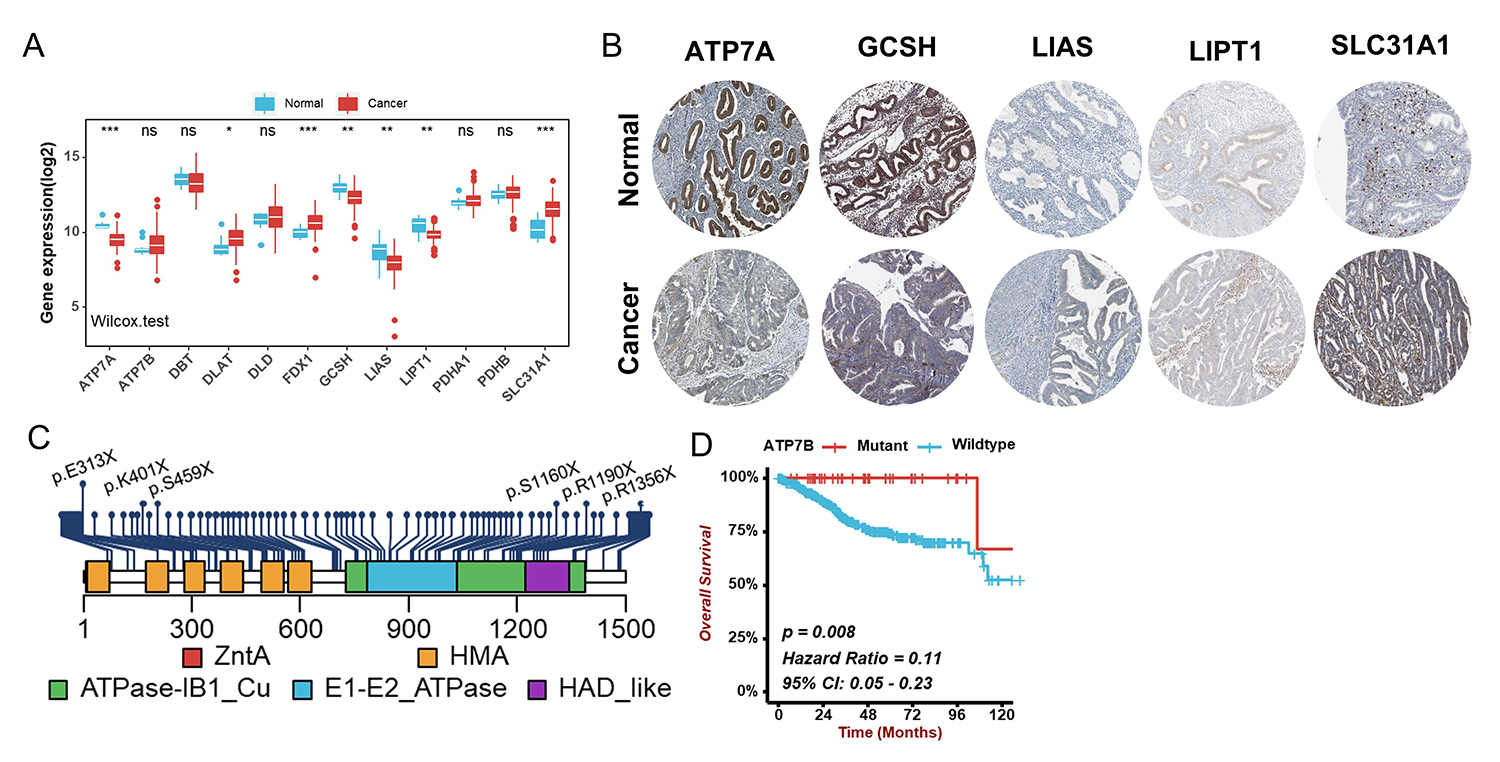

Supplement: Supplementary file 3 [file Image1.JPEG]

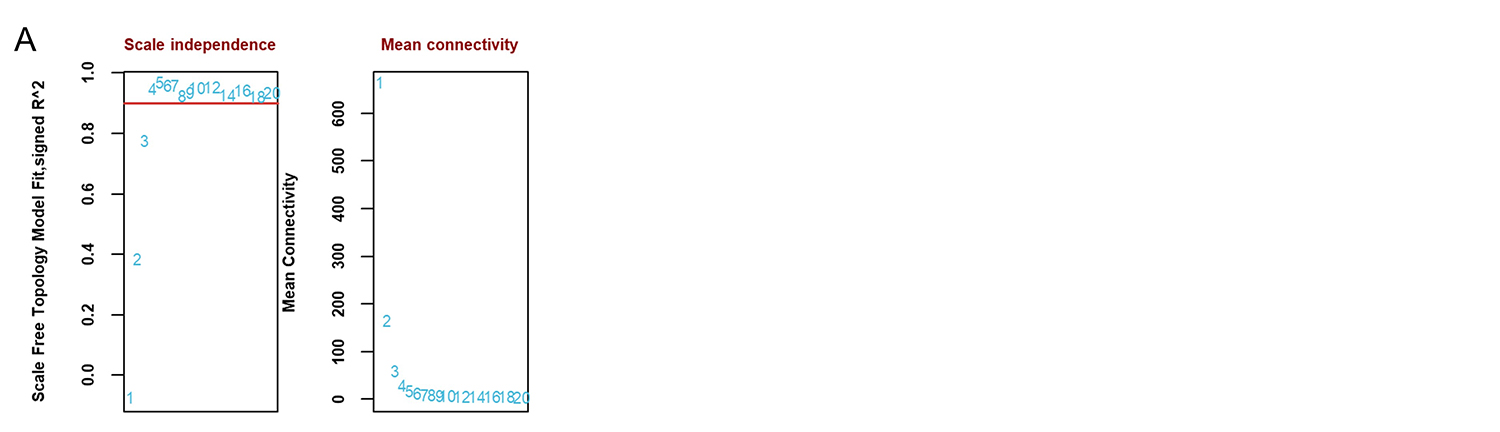

Supplement: Supplementary file 4 [file Image4.JPEG]

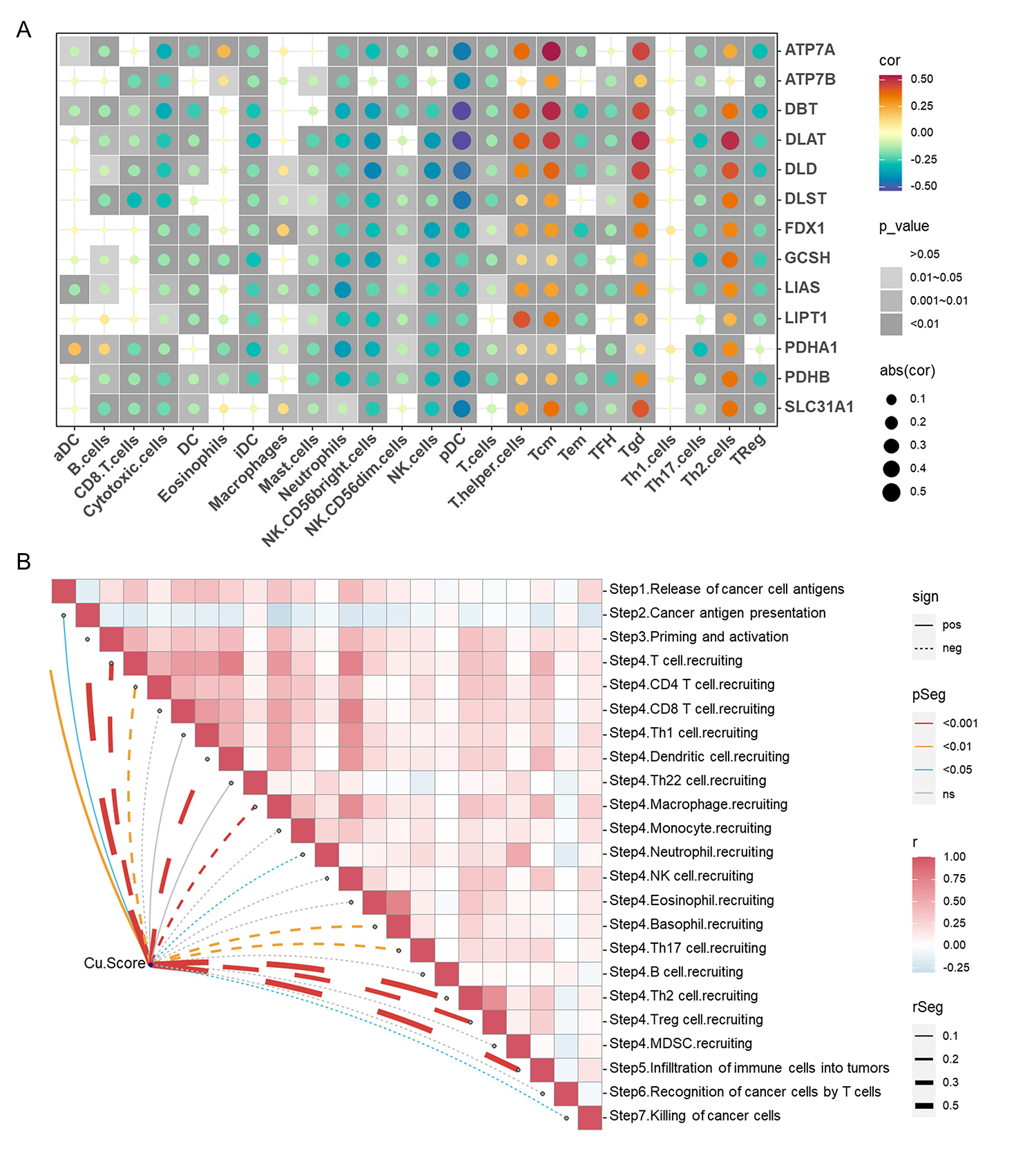

Supplement: Supplementary file 5 [file Image7.JPEG]

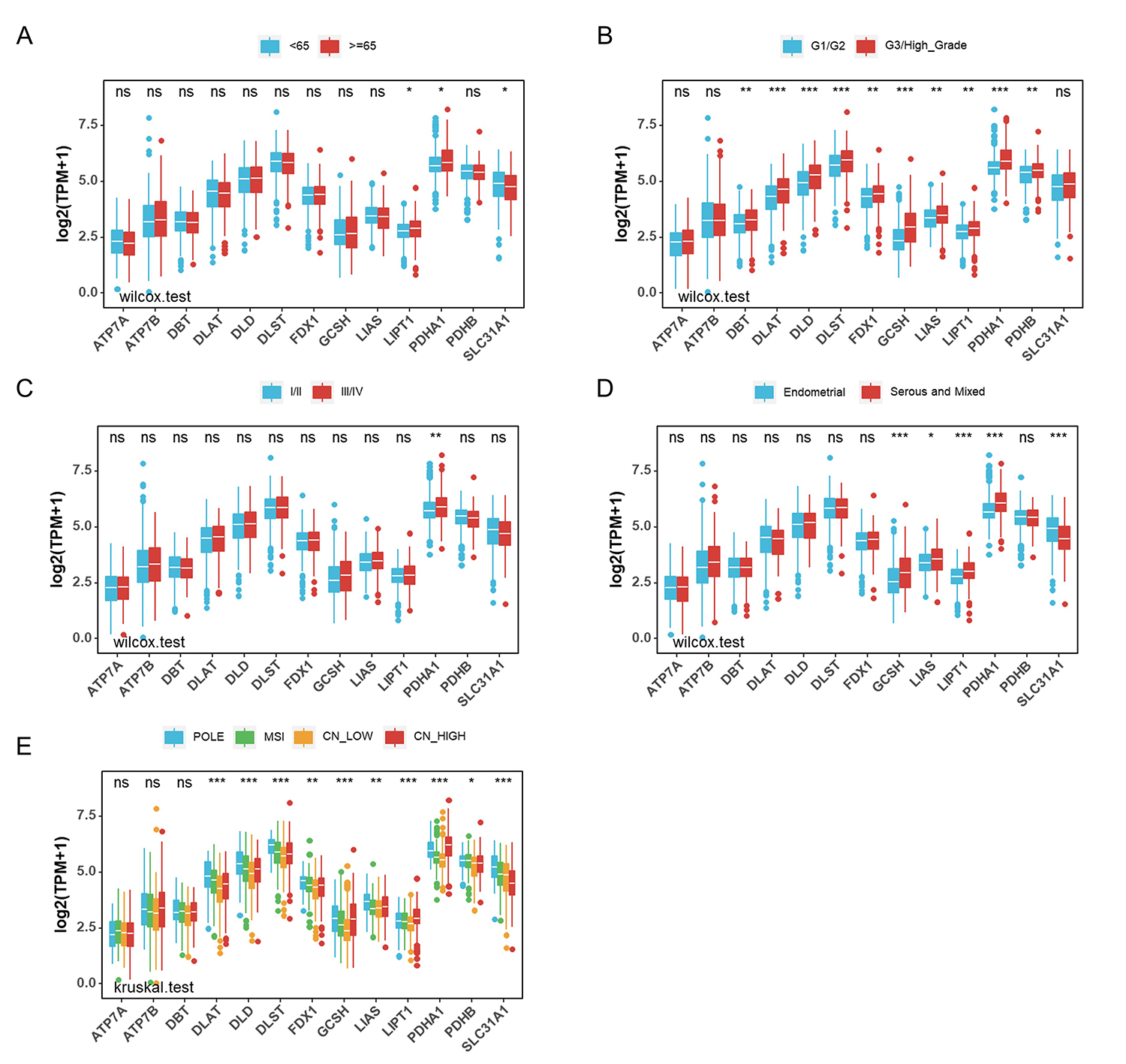

Supplement: Supplementary file 6 [file Image2.JPEG]

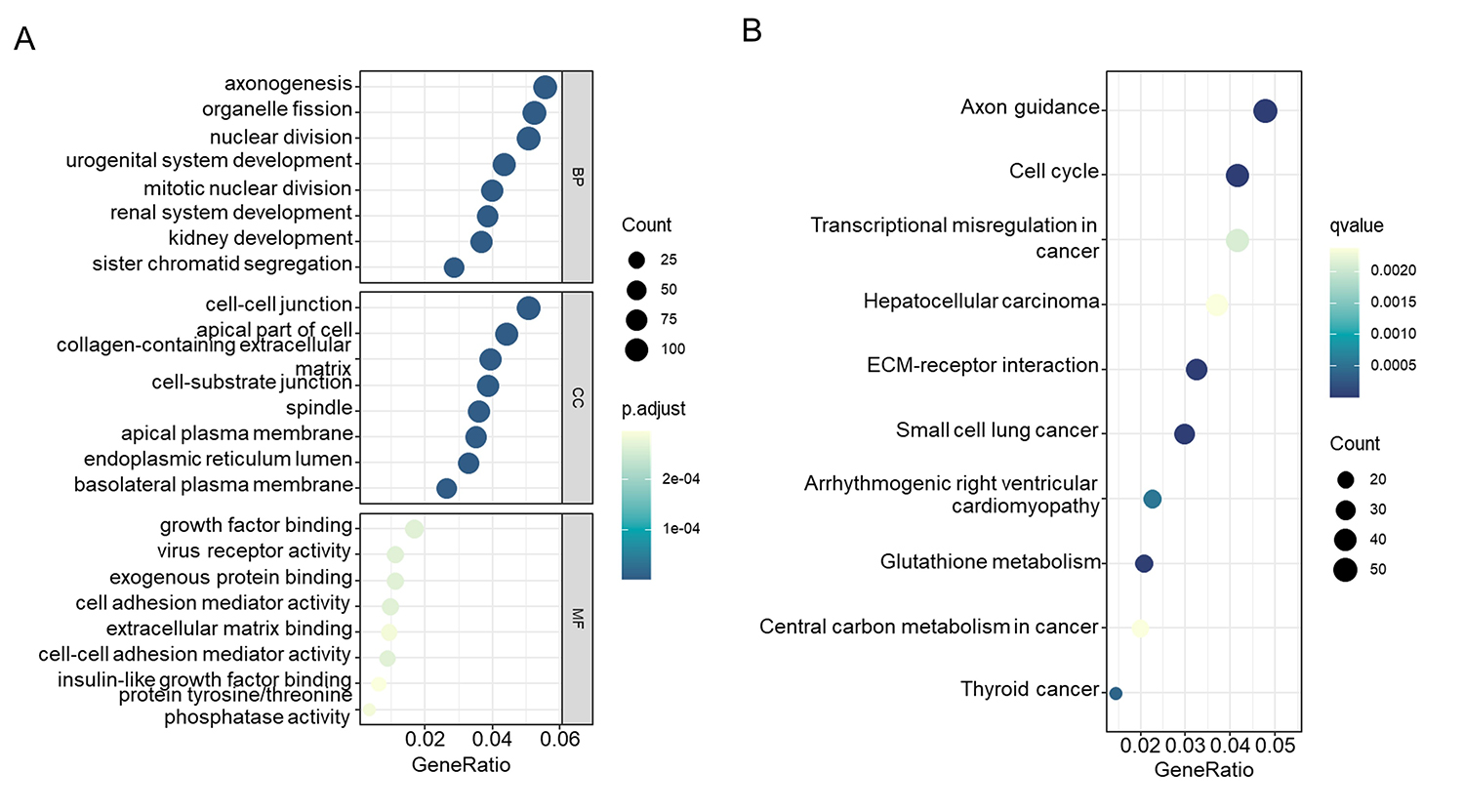

Supplement: Supplementary file 7 [file Image5.JPEG]

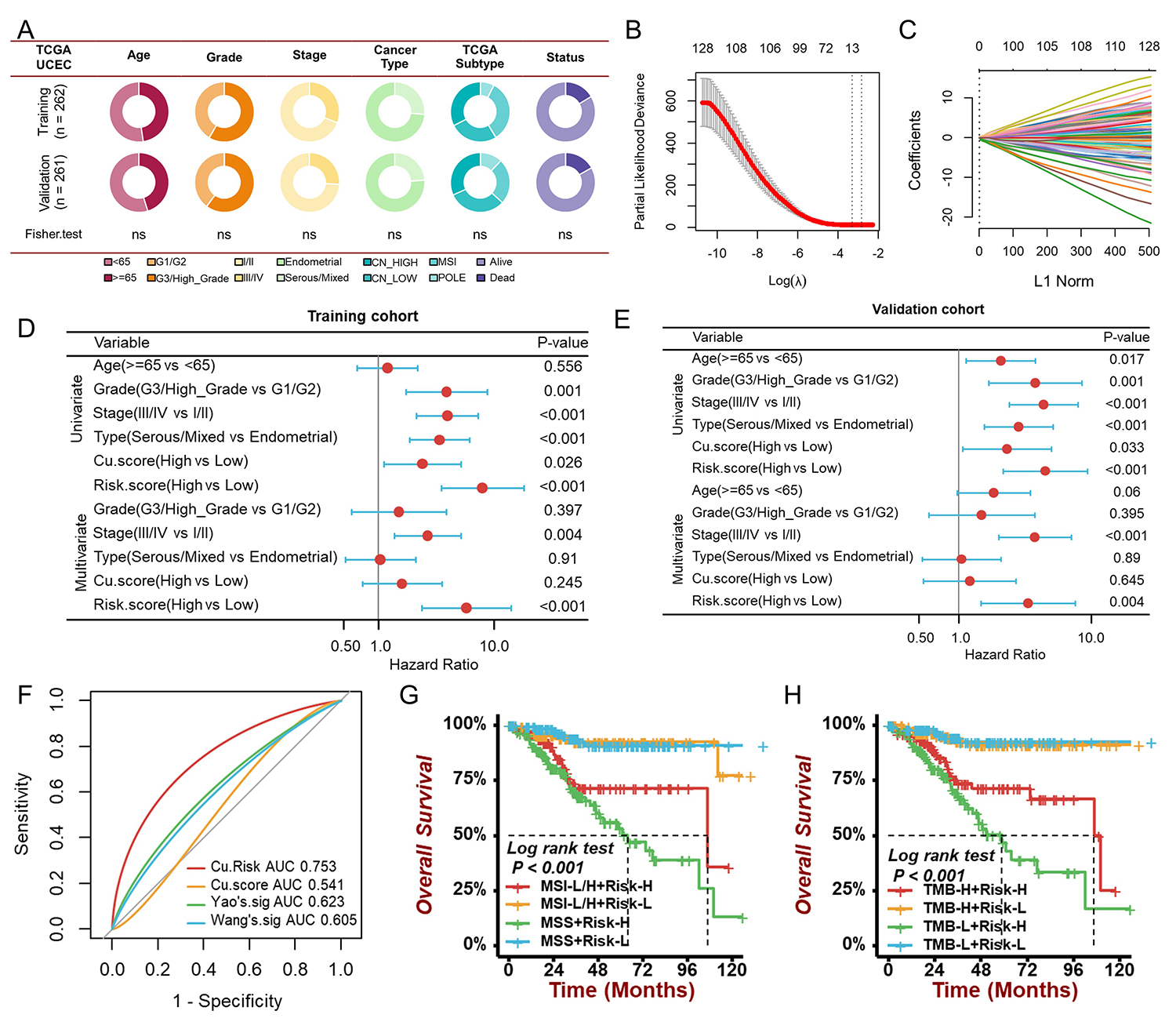

Supplement: Supplementary file 8 [file Image10.JPEG]

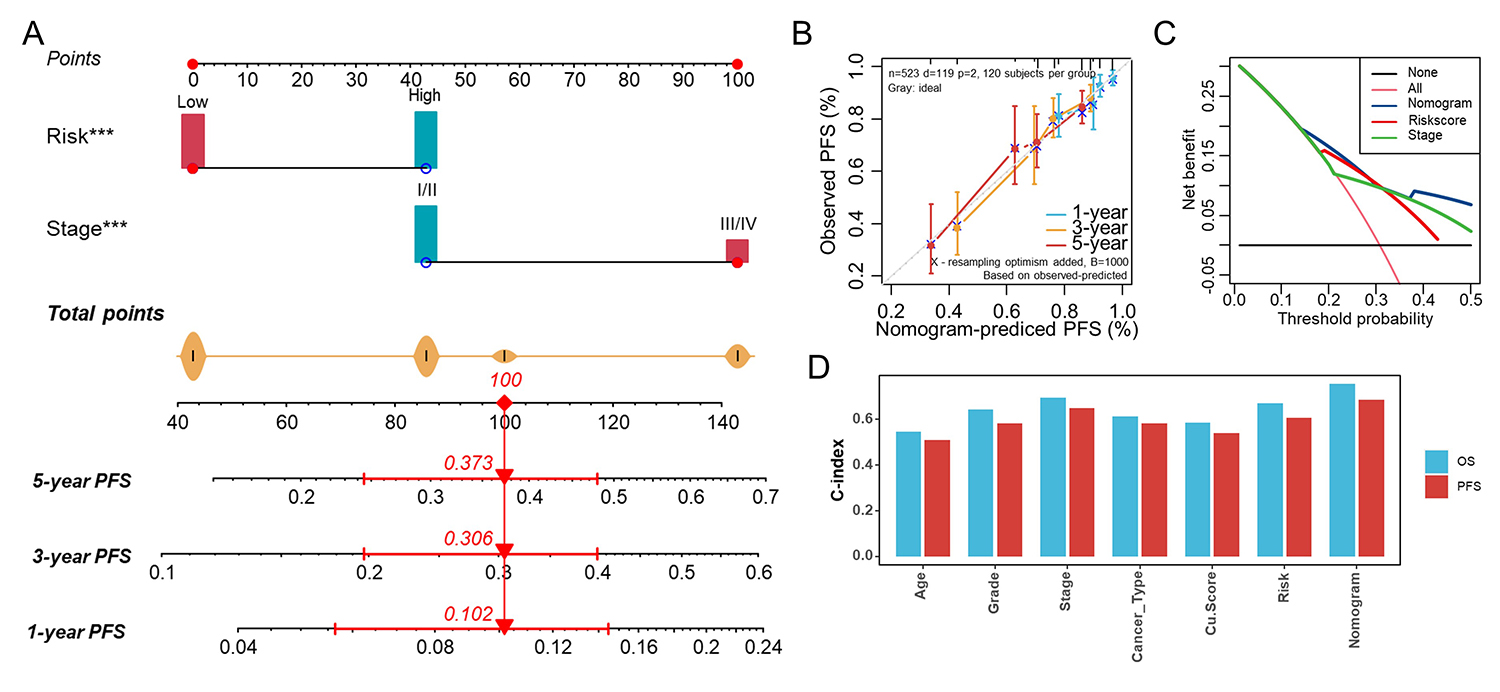

Supplement: Supplementary file 9 [file Image11.JPEG]

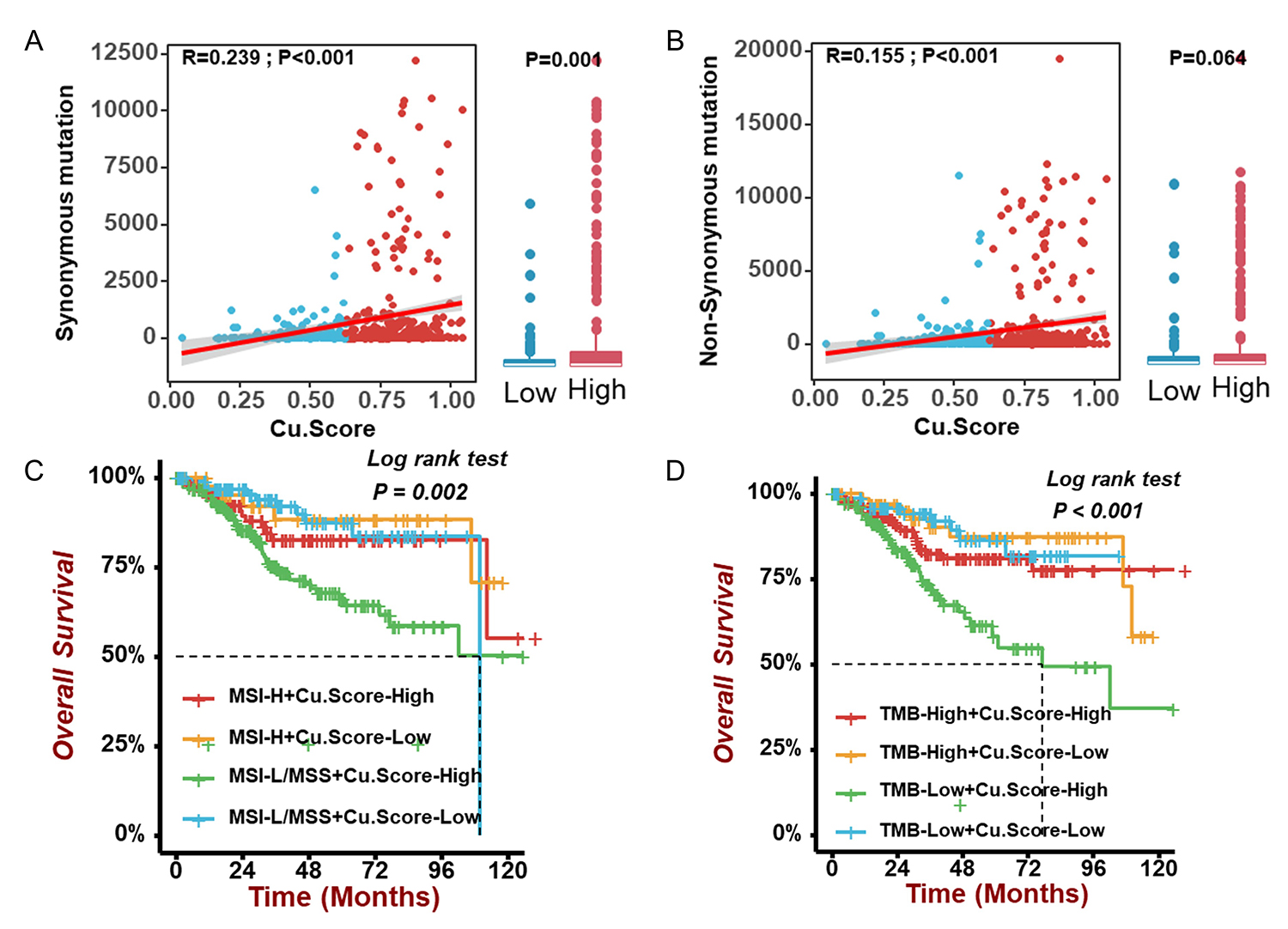

Supplement: Supplementary file 10 [file Image8.JPEG]

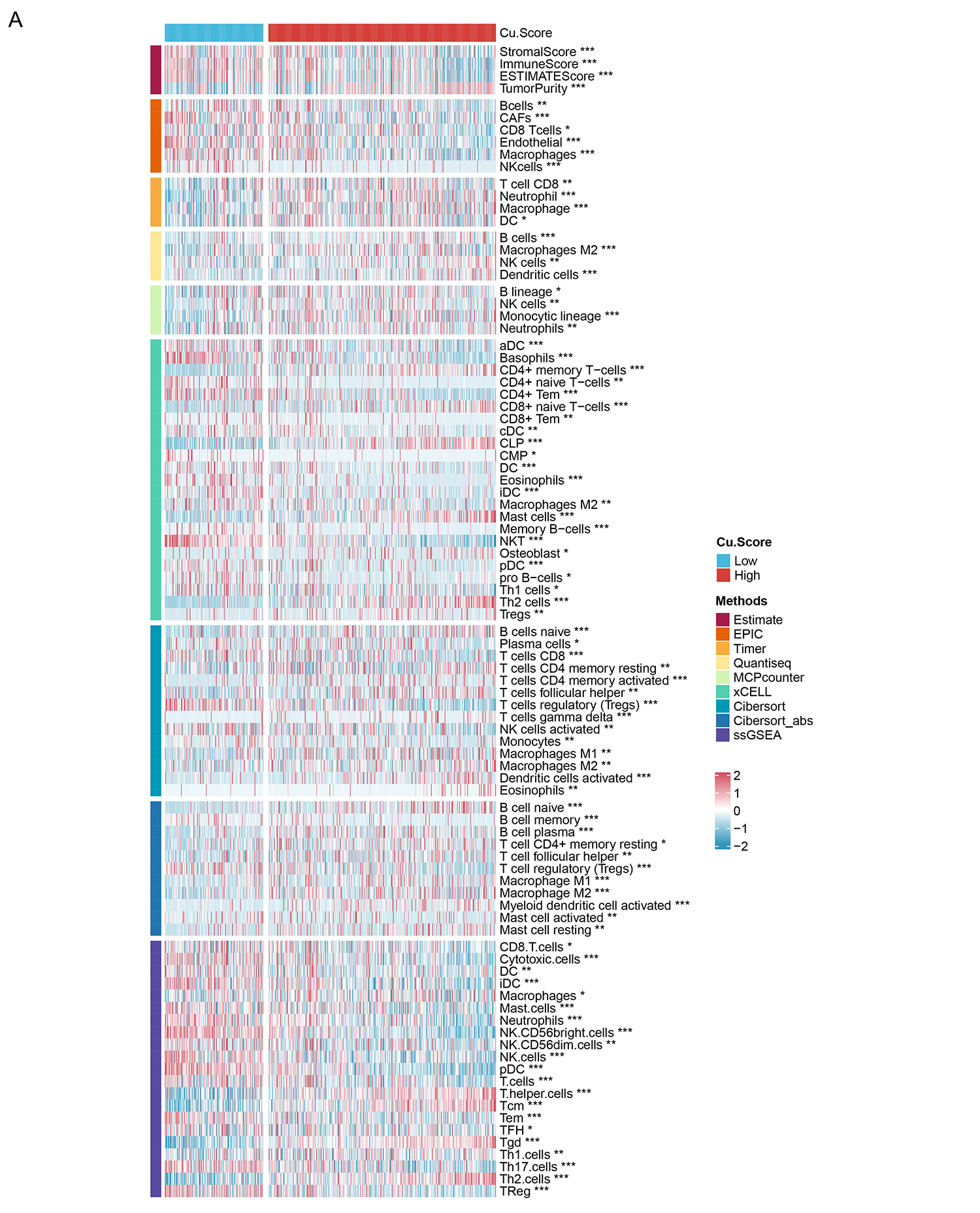

Supplement: Supplementary file 11 [file Image6.JPEG]
